# The Role of Social Isolation on Mediating Depression and Anxiety among Primary Family Caregivers of Older Adults: A Two‑Wave Mediation Analysis

**DOI:** 10.1007/s12529-023-10227-5

**Published:** 2023-10-25

**Authors:** Jiaming Liang, Maria P. Aranda, Yuri Jang, Kathleen Wilber

**Affiliations:** 1 Suzanne Dworak-Peck School of Social Work, University of Southern California, Los Angeles, USA; 2 Edward R. Roybal Institute on Aging, University of Southern California, Los Angeles, USA; 3 Department of Social Welfare, Ewha Womans University, Seoul, Republic of Korea; 4 Leonard Davis School of Gerontology, University of Southern California, Los Angeles, USA

**Keywords:** Family caregivers, Caregiving stress, Social isolation, Depression, Anxiety

## Abstract

**Background:**

Primary family caregivers of older people with chronic care conditions are highly vulnerable to social isolation and psychological strains such as depression and anxiety due to their demanding responsibilities. This study examines how social isolation mediates the relationship between caregiving stress and mental health symptoms of primary family caregivers.

**Methods:**

The analytic sample included 881 primary caregivers of older adults from the 2015 and 2017 National Study of Caregiving (NSOC). Social isolation was measured using a composite structure that includes objective social disconnectedness and subjective loneliness. Two-wave mediation models were estimated to examine longitudinally if social isolation mediated the relationship between caregiving stress (subjective & objective stress) and mental health symptoms (depression & anxiety) of primary caregivers.

**Results:**

The study findings indicate that both subjective (β = 0.32, p < 0.001) and objective stress (β = 0.21, p = 0.003) have direct effects on depression among primary caregivers. Social isolation was found to only mediate the relationship between objective stress and depression (β = 0.18, p < 0.001). In contrast, no significant direct and indirect pathway was found in the anxiety model.

**Conclusions:**

The study demonstrates the internal mechanism where objective strains of caregiving make family caregivers socially isolated, which in turn leads to increased symptoms of depression. Future interventions and practices aimed at improving the psychological well-being of family caregivers should prioritize strategies aimed at increasing social engagement, particularly for those with heavy caregiver burdens.

## Introduction

Globally, the aging population, particularly those with chronic health conditions and functional limitations, is creating an unprecedented demand for long-term care. In the United States, where the present study was conducted, approximately 80% of caregiving for older adults is provided by family caregivers, most of whom receive no compensation for their efforts [1]. Primary family caregivers, often partners or adult children, is the term for those who undertake most caregiving responsibilities and care tasks compared to secondary or tertiary caregivers [2, 3]. Previous research suggests that family caregivers are at risk for poorer emotional and mental health, sleep quality, and family relationships [4–6]. Primary caregivers tend to have higher care burden, mental distress, and financial stress, as well as lower quality of life than other family caregivers [7]. Therefore, it is critical to investigate the mechanisms by which caregiving stress impacts primary caregivers and identify ways to intervene the link between caregiving burden and emotional well-being.

Social isolation and loneliness are frequently reported among family caregivers. Caring for older adults with physical and cognitive limitations can cause chronic stress and caregiver burden often limits their social interactions. Research has linked social isolation to negative health outcomes, such as faster cognitive decline, increased depression, sleep disturbance, and higher mortality in older adults [8–10]. However, studies investigating the longitudinal effects of social isolation on family caregivers are limited, and there is a gap in the literature regarding whether social isolation can mediate the impact of caregiving stress on family caregivers’ mental health outcomes.

### Defining the Integrated Construct of Social Isolation

Despite the fact that some studies define social isolation as an objective measure that reflects the lack of social interaction and activity participation, and conceptually distinguish it from subjectively perceived loneliness [11, 12], it is essential to acknowledge that objective social contacts and activity participation may not fully reflect the quality of an individual’s social interactions. For example, older adults who participate in community activities may still experience significant loneliness due to widowhood. Similarly, despite that assuming caregiver role is associated with increased depression and loneliness, there is a stable or upward trend in the social network size of family caregivers [13, 14]. Thus, a composite scale for social isolation that includes objective and subjective indicators is necessary to comprehensively assess both the quantity and quality of an individual’s social situation.

This study adopted an integrated, multi-dimensional social isolation construct proposed by Cornwell and Waite in 2009, which includes objective social disconnectedness and subjective loneliness [15]. Objective social disconnectedness indicates lack of social interaction and participation in social activities, which has been associated with poor quality of life, physical disability, and cognitive impairment [15–17]. Subjective loneliness refers to a perceived inadequacy of the intimacy or companionship of one’s interpersonal relationships compared to the relationships that one would like to have [18]. Cornwell and Waite suggested that although the two dimensions of social disconnectedness and loneliness are not interchangeable, there is no theoretical reason to treat them as separate measures. An integrated construct of social isolation can improve the prediction of an individual’s physical and mental health outcomes [15].

### The Mediating Role of Social Isolation

Previous studies examined the role of social isolation on family caregivers. According to a recent review on social isolation of family caregivers [9], taking on the caregiver role reduces an individual’s social participation, and increases their level of social isolation. Moreover, social isolation among family caregivers is related to the functional limitations of the older adults they care for. Another study found that caregivers’ loneliness increased as participation in meaningful activities decreased due to caregiving tasks [19]. Notably, caregiving-related contextual factors have different effects on objective social disconnectedness and subjective loneliness. For example, although higher care demands and co-residing with care recipients may decrease caregivers’ social engagement and work-related time, the association between care demands and loneliness appears to be weaker [19, 20].

Such differences may be because different types of caregiving stress can affect the objective and subjective aspects of social isolation in distinct ways. Objective stress, such as care demands and caregiving workload, can directly affect family caregivers’ social participation and lead to social disconnection [19]. On the other hand, caregivers’ personal perceptions of the impact of caregiving activities on their own lives (subjective stress), may be influenced by their feelings of loneliness and the interactions they have with older adults they care for [21, 22]. Therefore, both types of stress–objective and subjective–need to be considered to accurately model the impact of caregiving stress on family caregivers’ social isolation.

The relationship between social isolation and family caregivers’ health has been extensively studied, with most studies indicating that social isolation has negative effects on caregivers’ sleep quality, mental health, and overall life satisfaction [23–25]. Research has also shown that socially disconnected family caregivers have poorer self-rated health, lower immunity, and more frequent hospital visits [26, 27], while subjective loneliness is associated with lower quality of life, feelings of helplessness, and more mental health symptoms such as depression and anxiety [9, 27]. Given the importance of maintaining strong social connections and social participation for the well-being of many family caregivers as well as for the sustainability of their caregiving duties, it is crucial to investigate the potential mediating role of social isolation in the relationship between caregiver stress and health outcomes.

### The Analytic Framework

This study used the Stress Process Model of Caregiving (SPM) as a guiding framework for the analysis. The SPM is a widely accepted theoretical model in family caregiving research that comprehensively describes the stress process from care tasks to care burden, as well as other psychological, social, and clinical health outcomes among family caregivers [28]. Reduced social and leisure engagement due to care responsibilities may eventually impact caregiver’s health [28]. The SPM also suggests that the accumulation and proliferation of caregiving stress have long-term effects on health [28, 29]. While previous studies have examined social isolation among family caregivers, these studies have primarily been cross-sectional, and there is a lack of research on the long-term mediation effects of social isolation on the relationship between caregiving stress and health outcomes. Thus, there is a critical need for longitudinal research to examine the mechanisms by which caregiving stress affects caregivers.

Taken together, social isolation may be an important secondary strain that mediates the impact of caregiving stress on the mental health symptoms of family caregivers of older adults. Therefore, the purpose of this study is to examine the mediating effect of social isolation on the association between two types of caregiving stress (i.e., objective and subjective stress) and mental health symptoms (i.e., depression and anxiety) among primary family caregivers. Our hypotheses are that (1) primary caregivers who experience higher levels of caregiving stress are more likely to be socially isolated, and (2) primary caregivers who are more socially isolated are more likely to report higher levels of mental health symptoms (i.e., depression and anxiety).

## Methods

### Participants

The present study utilized secondary data from the National Study of Caregiving (NSOC), a supplementary study to the National Health and Aging Trends Study (NHATS). NHATS is a survey that focuses on the health status, living conditions, and support environment of Medicare beneficiaries aged 65 and above [30]. Each NHATS participant identified up to 5 people who assisted them with mobility, self-care, or household activities to be interviewed for the NSOC regarding caregiving as well as personal, social, and health characteristics of caregivers [31]. The NHATS survey has been conducted annually since 2011; the NSOC interviews were conducted in 2011, 2015, and 2017 at the time of this analysis. The 2017 NSOC survey provided a longitudinal follow-up on the caregivers identified in 2015, allowing a longitudinal analysis of family caregivers using two waves of data.

The caregiver ID was linked to NHATS participants after merging the 2015 and 2017 NSOC data to identify older adults who met the inclusion criteria: (1) living in the community, (2) used no proxy for the interview, and (3) no missing data on key variables (e.g., functional limitations, cognitive status). The primary caregivers in the longitudinal sample were then identified using the following criteria: (1) nominated by older adults as primary caregiver; (2) providing the most caregiving hours compared to other nominated caregivers; (3) no missing data on key variables such as perceived care burden, social isolation, and mental health.

## Measures

### Mental Health Symptoms: Depression and Anxiety Symptoms

NSOC uses two short scales to assess depression and anxiety symptoms of caregivers (Patient Health questionnaire-2 [PHQ-2] and Generalized Anxiety Disorder scale-2 [GAD-2]). Both scales are widely used in public and mental health research [32, 33]. The items of the two scales are from two longer scales on depression and anxiety (PHQ-9 and GAD-7), and are considered to have the advantages of shorter space, time saving, and efficient screening [32, 33]. The PHQ-2 asks how often during the last month the respondent “had little interest or pleasure in doing things” and “felt down, depressed, or hopeless”; while the GAD-2 asks how often the respondent “felt nervous, anxious, or on edge” and “has been unable to stop or control worrying”. Each item of the two scales was measured from 1 (not at all) to 4 (nearly every day) with a total score ranges from 2 to 8. For both PHQ-2 and GAD-2, higher scores indicate more severe depression and anxiety.

### Caregiving Stress: Objective and Subjective Stress

Research has indicated that the experience of caregiving stress varies among caregivers from different racial/ethnic groups, potentially due to differences in cultural norms, family structures, and socioeconomic status [34]. Therefore, constructing latent variables in a structural equation model preserves the potential inequality of factor loadings across subgroups on the measure of caregiving stress, which may improve model efficiency over just using the composite score.

The latent factor of objective stress was built using two care recipient indicators: functional limitations and probable/possible dementia status. Functional limitations encompassed difficulties with activities of daily living (ADL) and instrumental activities of daily living (IADL) [35]. ADL includes dressing, bathing, eating, toileting, getting out of bed, and walking inside/outside home; IADL includes doing laundry, shopping, preparing meals, banking, and tracking medications. A ‘yes’ response to each item was scored as 1, and the sum score of these items ranges from 0 to 12, with higher scores indicating a greater level of functional limitations. The second indicator of objective stress was a binary variable representing older adults’ dementia status. The NHATS provides three criteria to identify probable/possible dementia of older adults, including (1) a dementia diagnosis from the doctor; (2) a score of 2 or more on AD-8 dementia screening; and (3) a score of 3 or more on the cognitive test on memory and orientation [36]. An older adult met any of the three criteria was considered to have probable/possible dementia.

The latent factor of subjective stress is based on financial, emotional, and physical strains reported by primary caregivers. Primary caregivers were specifically asked to rate the level of difficulty they experienced in each of these areas. Each strain was measured on a scale from 1 (a little difficult) to 5 (very difficult). The internal consistency (Cronbach’s α) of the three items was 0.69 in wave 1 and 0.73 in wave 2. Confirmatory factor analysis confirmed a two-factor measurement model with functional limitations (factor loading (FL) = 0.78) and dementia status (FL = 0.84) linked to objective stress, and the three strain variables associated with subjective stress, with factor loadings ranging from 0.72 to 0.80 (CFI = 0.991, TLI = 0.970, SRMR = 0.018, RMSEA = 0.041).

### Social Isolation

The measure of social isolation of primary caregivers used a composite framework that included objective social disconnectedness and subjective loneliness [15]. Objective social disconnectedness was computed using five items: having family/friends to talk with about important matters; having family/friends to visit; church participation; club participation; and volunteer work [26]. Each negative response to an item was scored as 1, and the total sum score of these items ranges from 0 to 5, with higher scores indicating greater objective social disconnectedness.

A single item loneliness scale was used to capture the subjective dimension of social isolation. Primary caregivers were asked “how often did you feel lonely during the last month?” Response ranged from 1 (never) to 5 (every day). The composite measure that integrated objective social disconnectedness and subjective loneliness exhibited adequate internal consistency (Cronbach’s α = 0.74 in wave 1 & 0.75 in wave 2) and excellent convergent validity in the measurement model with a single latent factor (Wave 1: RMSEA = 0.024, SRMR = 0.024, CFI = 0.985, TLI = 0.971; Wave 2: RMSEA = 0.031, SRMR = 0.022, CFI = 0.995, TLI = 0.989). The total score of social isolation ranged from 1 to 10, with higher scores indicating greater social isolation.

### Covariates

The analyses accounted for potential confounding factors that could be associated with mental health symptoms in caregivers based on the SPM [28], including sociodemographic characteristics, health status, and caregiving-related contextual factor of primary caregivers. Sociodemographic variables include age (in years), gender (man/woman), race (non-Hispanic White, non-Hispanic Black, Hispanic, others), and education level (<high school, high school, > high school). Health status included primary caregivers’ self-rated health (1–5) and number of chronic conditions (1–9: heart attack, other heart disease, hypertension, arthritis, osteoporosis, diabetes, lung disease, stroke, and cancer). A caregiving-related contextual factor, whether the primary caregiver co-resided with the older adult they care for, was included (yes/no).

### Statistical Analyses

Analyses were conducted using STATA/SE version 16.1 with a bilateral α = 0.05. Descriptive statistics were computed for all the variables: categorical variables were presented as frequency and percentage, and continuous variables as mean and standard deviation. To measure change overtime, McNemar’s χ^2^ and paired samples t-test were used to compare changes between the two waves. Correlations were computed for all variables to assess the risk of collinearity in the analysis models.

**Fig. 1:**
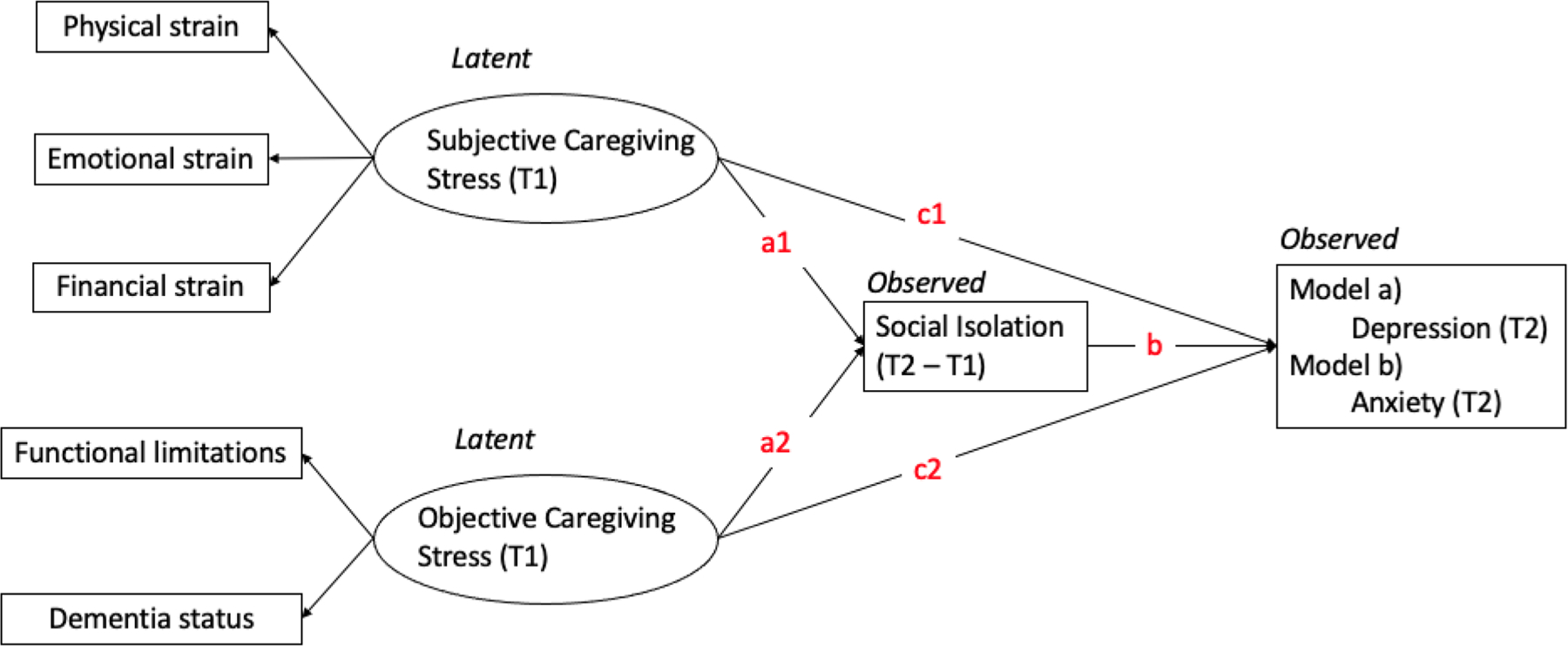
The analytic two-wave mediation model of the study

This study employed a longitudinal two-wave mediation analysis using structural equation modeling (SEM) to investigate whether the relationship between caregiving stress and the mental health symptoms of primary caregivers was mediated by social isolation [37, 38]. Two latent variables were created for objective and subjective caregiving stress, and separate models were fitted for depression and anxiety symptoms of primary caregivers (Fig. 1). The mediator was the change in primary caregivers’ social isolation between the two waves, calculated as the score differences. This approach overcomes the limitation of using only one wave of data as the mediator, preserving the predictive power of longitudinal data. Direct effects were the impact of the two caregiving stresses of wave 1 on the depression or anxiety of wave 2 (c1 & c2). Indirect effect refers to the impact of caregiving stress of wave 1 on the depression or anxiety of wave 2 through the score changes in social isolation between the two waves (a1*b & a2*b).

In the SEM models, only independent variables were treated as latent variables because: (1) The two indicators of objective stress were measured on different scales, making it impossible to add them up directly as observed scores; (2) The mediator was constructed using the score difference between the two waves, which cannot be operated on to create a latent variable. Also, factor analysis has already demonstrated the good convergent validity of this measure on a single latent factor; (3) The outcome variable utilized validated scales consisting of only two items. The model fit decreased after constructing a latent outcome variable, which suggests that the measurement errors of the two items may be correlated [39].

## Results

### Descriptive Statistics

The analytic sample consisted of 881 primary caregivers. Table 1 depicts the sociodemographic characteristics of the primary caregivers, and functional limitations and dementia status of older adults in 2015. Primary caregivers’ average age was 64 years old, with 49% being younger than 65 years old. Most are women (67%); 59% self-identified as non-Hispanic White. More than 85% of primary caregivers finished high school or have a higher degree. On average, each primary caregiver reported 1.85 (SD = 1.44) chronic conditions; the three conditions with the highest prevalence were hypertension (54%), arthritis (45%), and diabetes (22%). The prevalence of all other conditions was below 15%. Care recipients reported an average of 6.41 (SD = 3.10) functional limitations out of 12 items, with 41% having possible/probable dementia.

A comparison was conducted to examine the distribution of variables among participants of different races/ethnicities (i.e., non-Hispanic White, non-Hispanic Black, Hispanic, and others). Compared to White participants, Black caregivers have younger age (t = 5.22, p < 0.001), lower educational level (χ^2^ = 32.51, p < 0.001), are more likely to be unmarried (χ^2^ = 59.97, p < 0.001), and to take care for their own parents (χ^2^ = 36.06, p < 0.001). The cumulative scores of social isolation were not significantly different between the groups, though Black caregivers reported higher prevalence of having no family/friend to visit (χ^2^ = 11.64, p = 0.001), no club participation (χ^2^ = 5.58, p = 0.02), and no volunteer work (χ^2^ = 12.01, p = 0.001). Similar patterns were observed within the Hispanic group. Participants of other racial/ethnic group showed similar distributions across most variables compared to non-Hispanic White, albeit displaying a significantly higher level of social isolation (t = 2.90, p = 0.002). Detailed comparison results are listed in Supplementary Table 1.

Table 2 presents comparisons of key variables between the two waves. In wave 1, the mean of self-rated primary caregivers’ health was 3.41 (SD = 1.10) on a 1 to 5 scale, which decreased significantly to 3.32 (SD = 1.09) in wave 2 (t = 3.18, p < 0.01). Subjectively perceived caregiving strain also significantly increased from an average of 4.79 (SD = 2.62) on a 3 to 15 scale in wave 1, to 5.00 (SD = 2.85) in wave 2 (t = 2.49, p < 0.05). Both emotional and physical difficulty increased significantly, while financial difficulty did not change. The overall score for social isolation increased from 3.69 (SD = 1.71) in wave 1 to 4.26 (SD = 1.94) in wave 2 (t = 9.50, p < 0.001). Except for club participation, primary caregivers’ responses to all other indicators of objective social disconnectedness changed significantly between the two waves. The proportion of participants reporting no friend/family to talk to in wave 2 (31.56%) was almost double than that of wave 1 (16.91%; χ^2^ = 49.88, p < 0.001). The proportion of visiting no friend/family increased from wave 1 (23.04%) to wave 2 (34.17%; χ^2^ = 34.55, p < 0.001), the proportion of no church participation increased from wave 1 (39.50%) to wave 2 (50.17%; χ^2^ = 38.75, p < 0.001), and the proportion of no volunteer work also increased from wave 1 (76.17%) to wave 2 (82.97%; χ^2^ = 23.68, p < 0.001). Similarly, subjective loneliness scores increased significantly from 1.85 (SD = 0.96) in wave 1 to 1.96 (SD = 1.09) in wave 2 (t = 3.28, p < 0.01). For mental health symptoms, primary caregivers reported a mean depression score of 3.08 (SD = 1.36), which increased significantly to 3.42 (SD = 1.57) in wave 2 (t = 6.65, p < 0.001). Similarly, the mean anxiety score increased from 3.07 (SD = 1.39) in wave 1 to 3.16 (SD = 1.48) in wave 2 (t = 2.18, p < 0.05). Correlation analysis revealed that none of the coefficients between variables exceed 0.4, indicating a low risk of collinearity in analytic models (see Supplementary Table 2).

### The Mediating Effects of Social Isolation

#### Depression Model

**Fig. 2:**
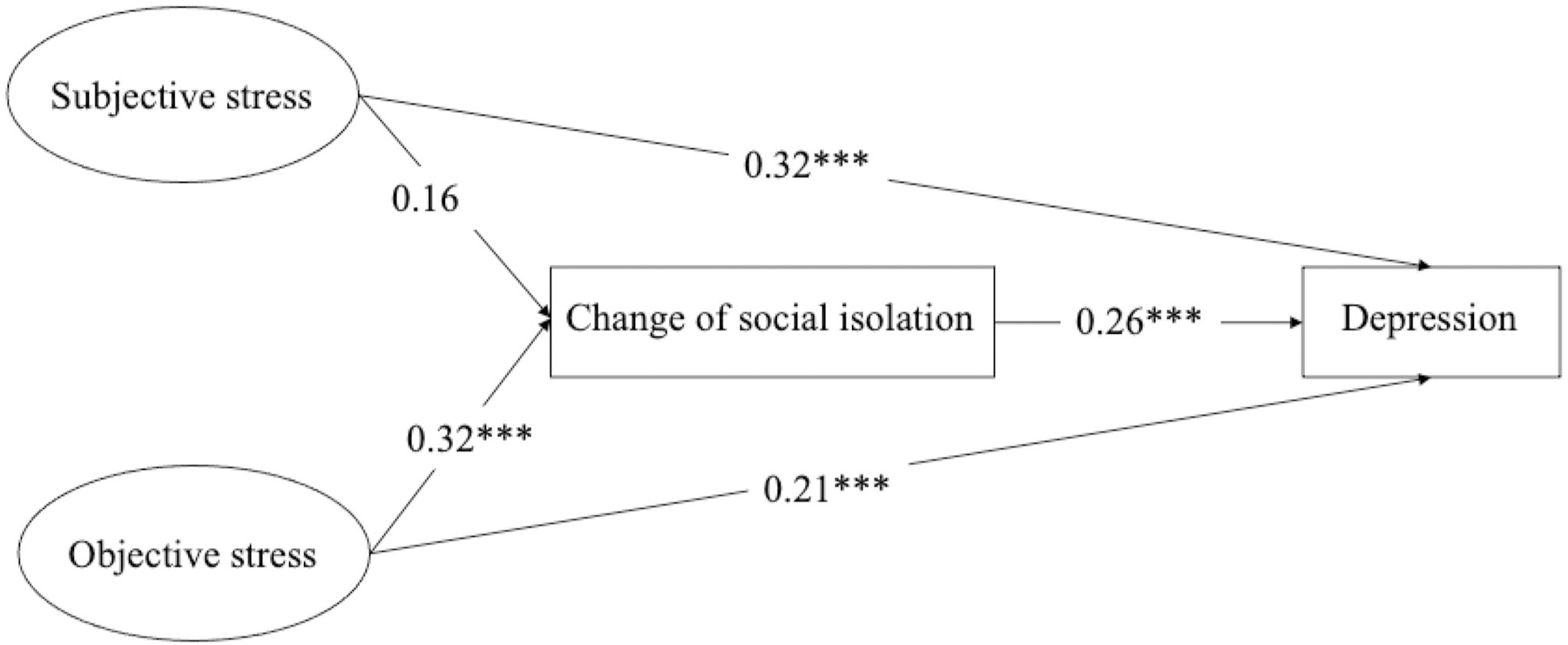
The mediated model of social isolation between caregiving stress and depression in primary caregivers

Table 3 and Fig. 2 present the results of SEM on depression. Findings of the two-wave mediation model indicate a mediated path connecting objective caregiving stress and depression through social isolation (β = 0.18, se = 0.03, p < 0.001). This suggests that increased levels of objective caregiving stress led to higher social isolation among primary caregivers, which then exacerbated their depression. However, the impact of subjective caregiving stress on depression was not mediated by social isolation (β= 0.04, se = 0.03, p= 0.18), because the increase of subjective caregiving stress did not result in a deterioration of social isolation (β = 0.16, se = 0.12, p = 0.17).

The mediated effect of social isolation partially explains the impacts of the two types of caregiving stress on the depression of primary caregivers. Specifically, subjective stress in wave 1 was directly related to depression in wave 2 (β = 0.32, se = 0.09, p < 0.001), as was objective stress (β = 0.21, se = 0.07, p < 0.01). This partially supports the study hypotheses that social isolation only mediated the relationship between objective caregiving stress and depression of primary caregivers. The model demonstrates good fit (RMSEA = 0.028, SRMR = 0.020, CFI = 0.990, TLI = 0.980). Regarding covariates, caregivers from other racial/ethnic groups tend to exhibit lower levels of depression than non-Hispanic White (β = −0.59, se = 0.21, p = 0.005), those who reported better self-rated health at wave 1 tend to report reduced depression in wave 2 (β = −0.27, se = 0.05, p < 0.001), while living together with the individuals they care for is associated with higher levels of depression (β = 0.28, se = 0.12, p < 0.001).

#### Anxiety Model

A second model was estimated to examine if social isolation was a mediator between caregiving stress and anxiety of primary caregivers (Table 4). Findings indicate that social isolation did not mediate the impact of subjective stress (β = 0.01, se = 0.08, p = 0.68) or objective stress (β = 0.27, se = 0.29, p = 0.39) on the anxiety level of primary caregivers.

For each individual path, only objective stress to social isolation is significant (β = 0.32, se = 0.09, p < 0.001), which is consistent with the results of the depression model. None of the remaining paths are significant, suggesting that neither subjective nor objective stress had an impact on anxiety of primary caregivers (RMSEA = 0.063, SRMR = 0.043, CFI = 0.904, TLI = 0.886). Regarding covariates, Black caregivers tend to have lower level of anxiety than non-Hispanic White (β = −0.24, se = 0.11, p = 0.03). Similar to the depression model, participants with better self-rated health tend to report lower level of anxiety (β = −0.26, se = 0.05, p < 0.001).

## Discussion

This study investigated a stress process mediated by social isolation in primary family caregivers of older adults, using two nationally representative datasets. An integrated measurement of family caregivers’ social isolation was used, encompassing both objective social disconnectedness and subjective loneliness. The utilization of longitudinal data facilitated the construction of a two-wave mediation model, which examined the impact of caregiving stress on the mental health symptoms (depression and anxiety) of family caregivers, mediated by social isolation. Although the study focused exclusively on primary caregivers, who often bear the highest burden and experience the most significant mental strain, the findings may also have implications for other family caregivers who do not hold the primary caregiving role. Overall, the findings partially support the study hypotheses, specifically that objective caregiving stress increased social isolation of family caregivers, which consequently increased depressive symptoms.

The results of the comparison using two waves of data revealed that primary caregivers experienced significant increases in social isolation within the two-year period. This supports previous research that assuming the role of family caregiver impacts one’s social network, reduces social connection and participation, and increases loneliness [9, 19]. The most substantial changes occurred in the frequency of interaction with friends and family members, which might imply a decrease of social support that family caregivers can access, and a heightened risk of mental health issues [40]. Another significant change was the reduced involvement in church activities. Church activities often require a fixed amount of time, such as morning hours, as compared to other social activities, such as clubs or volunteer activities, that offer greater flexibility regarding timing, thus allowing participants to adjust their schedules accordingly. Previous research highlights the centrality of religious activities in mental health through providing psychological protection, meaning and purpose in life, and expanding social networks and support systems [41, 42]. Consequently, reduced attendance at church activities may have a substantial impact on caregivers’ mental health.

Consistent with prior research, primary caregivers experienced significant increase in depression and anxiety levels [43, 44]. Caring for an older adult with functional needs increases stress, and caregivers’ depressive symptoms may be exacerbated by the continual sacrifice of time and energy, as well as frustration with caregiving tasks [44]. In comparison, anxiety levels showed a smaller change than depression, possibly because that caregiving-related anxiety is primarily a feeling of being overwhelmed, resulting from caregiving tasks that exceed caregivers’ coping capacity [45]. Family caregivers may improve their caregiving skills and coping capacity over time, or seek skilled assistance with caregiving tasks to alleviate anxiety levels. However, family caregivers’ depressive symptoms may persist longer. One study found that depressive symptoms can last for at least a year even after cessation of the caregiving role [46].

The findings showed that social isolation mediated the relationship between objective caregiving stress and depression among primary caregivers, accounting for 28.4% of the total impact of objective stress on depression. This supports the notion that reduced social engagement can compromise emotional stability [44, 45]. Although subjective caregiving stress also was found to have a direct effect on depression, no significant mediated effect of social isolation was observed in this path. Objective stress, which reflects caregiving workload and the time and effort family caregivers devote to caregiving, is different from subjective stress, which involves an appraisal process that considers the caregiver’s resilience and available social support resources [19, 22]. Family caregivers who experience social isolation or have limited access to social support may perceive higher levels of caregiving stress than those with relatively adequate support resources [17, 19]. Therefore, higher subjective stress may result from being socially isolated, rather than the reverse.

The analyses revealed no significant mediated effect in the anxiety model, and the total effect of caregiving stress at wave 1 on primary caregivers’ anxiety at wave 2 was not significant. This may be attributed to the previously discussed differences in the underlying mechanisms of depression and anxiety in family caregivers. Specifically, caregiver’s anxiety occurs when the demands of caregiving tasks exceeds the family caregiver’s coping capacity [45], and can be mitigated by enhancing caregivers’ skills and self-competence, as well as by receiving hands-on assistance with caregiving tasks [47]. Future research should adopt shorter intervals to capture the changing dynamics of family caregivers’ anxiety.

A major methodological strength of this study is that it uses a longitudinal perspective to explore the social isolation and mediated stress process among family caregivers. The two-wave mediation model examines causal relationships based on two waves of data [37, 38]. In addition, the study sample is from nationally representative datasets with distributions of key variables consistent with national estimations from NHATS and NSOC [48], such as proportion of women caregivers and probable/possible dementia prevalence among older adults. Thus, our sample has a low risk of decreased representativeness due to the data screening.

This study has some limitations. First, the two-year interval used in this longitudinal design might be too long to capture dynamic changes of mental health symptoms of participants. Future research with shorter intervals and multiple waves may provide more accurate insights. Second, the measurement of social isolation used in this study was constructed based on theoretical frameworks, and its reliability and validity among the population of caregivers have not been tested. While factor analyses did provide support for its validity, more research is needed to validate this measurement. Third, this study only estimated models for depression and anxiety. Other metrics related to mental health and well-being of family caregivers, such as subjective happiness, satisfaction, and resilience, should be investigated in the future. Last, socio-demographics of family caregivers (e.g., race/ethnicity, gender, education, income, etc.) can shape their ability to afford professional caregiving assistance and engage in social activities, thus impacting their overall stress experience. While this study conducted supplementary analyses to compare variable distributions across racial/ethnic groups, it is important for future research to concurrently consider other socioeconomic factors and their potential intersections.

This study has important implications for community-based practice and intervention development. Social isolation can partially explain the impacts of caregiving stress on family caregivers’ depression, which highlights the importance of interventions aimed at promoting caregivers’ social connection and reducing loneliness. Existing intervention programs mostly provide psychoeducation courses regarding health literacy and caregiving skills, as well as connect caregivers with formal services, refer to professional assistance, and provide assistive technology [49–51]. Only a few address the social isolation of caregivers. For example, one program used digital tools to facilitate informal social support, which signaled potential in addressing the social needs of caregivers who are unable to participate in in-person activities [52, 53]. Future research should explore the effectiveness of web-based social participation in compensating for the social needs of family caregivers.

## Conclusion

This study employed a longitudinal design to investigate the mediated effect of social isolation on the relationship between caregiving stress and mental health symptoms of primary caregivers of older adults. The findings suggest that caregiving stress may have a negative impact on the mental well-being of family caregivers by impeding their social ties and activity participation, such as visiting family or friends and attending religious activities, as well as heightening their feelings of loneliness. Interventions aimed at alleviating caregiving burden and protecting mental health should focus on encouraging the utilization of family and community resources to mitigate care intensity, and fostering meaningful social network engagement and activity participation among family caregivers.

## Supplementary Material

Supplementary Tables 1 & 2

## Figures and Tables

**Table 1:** The descriptive characteristics of primary caregivers and older adults in 2015 (N = 881)

		Frequency	%	Mean	SD
*Primary caregivers*					
Age				64.09	13.08
	< 55	196	22.25%		
	55—64	234	26.56%		
	65—74	236	26.79%		
	75 -	205	23.27%		
Gender	Man	289	32.80%		
	Woman	592	67.20%		
Race/Ethnicity	Non-Hispanic White	518	58.80%		
	Non-Hispanic Black	164	18.62%		
	Hispanic	101	11.46%		
	Others	98	11.12%		
Education	Below high school	128	14.53%		
	High school no college	448	50.85%		
	Bachelor and above	305	34.62%		
Marital Status	Married/with partner	558	63.34%		
	Unmarried	323	36.66%		
Relationship Type	Spouse/Partner	300	34.05%		
	Adult Child	451	51.19%		
	Others	130	14.76%		
Co-residence	No	320	36.32%		
	Yes	561	63.68%		
No. of Chronic Conditions				1.85	1.44
*Older adults*					
Functional Limitations				6.41	3.10
Probable/Possible Dementia (yes)		358	40.64%		

**Table 2:** The comparisons of key variables between the two waves (N = 881)

	Wave 1 (2015)					Wave 2 (2017)				McNemar’s χ^2^ / (Paired t)
	Frequency	%	Mean	SD		Frequency	%	Mean	SD	
Self-rated health			3.41	1.10				3.32	1.09	(3.18**)
Perceived difficulty			4.79	2.62				5.00	2.85	(2.49*)
Financial difficulty			1.43	1.02				1.42	1.05	(0.21)
Emotional difficulty			1.83	1.26				1.97	1.40	(2.92**)
Physical difficulty			1.53	1.14				1.62	1.19	(2.20*)
Social isolation			3.69	1.71				4.26	1.94	(9.50***)
Social disconnectedness			2.18	1.27				2.63	1.64	(7.63***)
No friend/family to talk	149	16.91%				278	31.56%			49.88***
No visit to friend/family	203	23.04%				301	34.17%			34.55***
No church participation	348	39.50%				442	50.17%			38.75***
No club participation	594	67.42%				615	69.81%			1.85
No volunteering	671	76.17%				731	82.97%			23.68***
Loneliness			1.85	0.96				1.96	1.09	(3.28**)
Depression			3.08	1.36				3.42	1.57	(6.65***)
Anxiety			3.07	1.39				3.16	1.48	(2.18*)

* p < 0.05

** p < 0.01

*** p < 0.001

**Table 3:** The mediated model of social isolation between caregiving stress and depression in primary caregivers

	β (se)	p
Direct paths		
Change of social isolation (Mediator)		
Subjective stress	0.16 (0.12)	0.17
Objective stress	0.32 (0.09)	< 0.001
Depression (Dependent variable)		
Change of social isolation	0.26 (0.03)	< 0.001
Subjective stress	0.32 (0.09)	< 0.001
Objective stress	0.21 (0.07)	0.003
Indirect paths		
SS^a^ – > CSI^b^ – > Depression	0.04 (0.03)	0.18
OS^c^ – > CSI – > Depression	0.18 (0.03)	< 0.001
Covariates (primary caregiver characteristics)		
Age	0.001 (0.004)	0.77
Gender (women)	−0.01 (0.1)	0.91
Race (non-Hispanic White as reference)		
Non-Hispanic Black	0.07 (0.11)	0.51
Hispanic	−0.11 (0.21)	0.59
Others	−0.59 (0.21)	0.005
Relationship type (spouse as reference)		
Adult child	0.04 (0.15)	0.81
Others	−0.06 (0.19)	0.77
Self-rated health	−0.27 (0.05)	< 0.001
Number of chronic conditions	0.05 (0.04)	0.19
Co-residence	0.28 (0.12)	0.02

Model was adjusted for primary caregivers’ age, gender, race, relationship type, self-rated health, number of chronic conditions, and coresidential status

a SS: Subjective Stress

b CSI: Change of Social Isolation

c OS: Objective Stress

**Table 4:** The mediated model of social isolation between caregiving stress and anxiety in primary caregivers

	β (se)	p
Direct paths		
Change of social isolation (mediator)		
Subjective stress	0.31 (0.23)	0.18
Objective stress	0.32 (0.09)	< 0.001
Anxiety		
Isolation change	0.07 (4.03)	0.98
Subjective stress	0.53 (0.82)	0.06
Objective stress	−0.09 (0.72)	0.98
Indirect paths		
SS^a^ – > CSI^b^ – > Anxiety	0.01 (0.08)	0.68
OS^c^ – > CSI – > Anxiety	0.27 (0.29)	0.39
Covariates (primary caregiver characteristics)		
Age	−0.01 (0.005)	0.09
Gender (women)	0.1 (0.1)	0.32
Race (non-Hispanic White as reference)		
Non-Hispanic Black	−0.24 (0.11)	0.03
Hispanic	0.02 (0.21)	0.93
Others	−0.41 (0.21)	0.06
Relationship type (spouse as reference)		
Adult child	−0.18 (0.15)	0.22
Others	−0.29 (0.19)	0.12
Self-rated health	−0.26 (0.05)	< 0.001
Number of chronic conditions	0.04 (0.04)	0.37
Co-residence	−0.05 (0.12)	0.66

Model was adjusted for primary caregivers’ age, gender, race, relationship type, self-rated health, number of chronic conditions, and coresidential status

a SS: Subjective Stress

b CSI: Change of Social Isolation

c OS: Objective Stress

## Data Availability

This study used a public dataset that can be accessed at: https://www.nhats.org/researcher/nsoc.
