# Supplementary material for: The Role of Social Isolation on Mediating Depression and Anxiety among Primary Family Caregivers of Older Adults: A Two‑Wave Mediation Analysis
Source: Int J Behav Med. Author manuscript; Available in PMC 2024 Jun 1. (PMC11043211; doi:10.1007/s12529-023-10227-5)
Supplement: Supplementary Tables 1 & 2 [file NIHMS1942543-supplement-Supplementary_Tables_1___2.docx]

Supplementary Table 1. Comparisons of variables across racial/ethnic groups

|  |  | Non-Hispanic White  (N = 518, 59%) | | Non-Hispanic Black  (N = 164, 19%) | | | Hispanic  (N = 101, 11%) | | | Others  (N = 98, 11%) | | |
| --- | --- | --- | --- | --- | --- | --- | --- | --- | --- | --- | --- | --- |
|  |  | Mean (SD) | % | Mean (SD) | % | p | Mean (SD) | % | p | Mean (SD) | % | p |
| Age |  | 66.3 (12.28) |  | 61.31 (13.07) |  | <0.001 | 55.32 (15.97) |  | <0.001 | 64.51 (11.73) |  | 0.99 |
|  | < 55 |  | 17.28% |  | 29.34% | <0.001 |  | 42.00% | <0.001 |  | 21.28% | 0.78 |
|  | 55 - 64 |  | 24.08% |  | 32.43% |  |  | 26.00% |  |  | 27.66% |  |
|  | 65 - 74 |  | 30.49% |  | 20.46% |  |  | 26.00% |  |  | 27.66% |  |
|  | 75 - |  | 28.16% |  | 17.76% |  |  | 6.00% |  |  | 23.40% |  |
| Gender | Men |  | 33.98% |  | 30.30% | 0.30 |  | 37.25% | 0.64 |  | 29.17% | 0.50 |
|  | Women |  | 66.02% |  | 69.70% |  |  | 62.75% |  |  | 70.83% |  |
| Education | Below high school |  | 9.07% |  | 23.11% | <0.001 |  | 23.53% | 0.001 |  | 16.67% | 0.23 |
|  | High school no college |  | 51.35% |  | 49.62% |  |  | 54.90% |  |  | 47.92% |  |
|  | Bachelor and above |  | 39.58% |  | 27.27% |  |  | 21.57% |  |  | 35.42% |  |
| Marital Status | Married/with partner |  | 73.55% |  | 45.45% | <0.001 |  | 50.98% | 0.001 |  | 64.58% | 0.18 |
|  | Unmarried |  | 26.45% |  | 54.55% |  |  | 49.02% |  |  | 35.42% |  |
| Relationship Types | Spouse/Partner |  | 41.31% |  | 21.97% | <0.001 |  | 21.57% | 0.007 |  | 35.42% | 0.69 |
|  | Adult child |  | 47.88% |  | 56.06% |  |  | 56.86% |  |  | 54.17% |  |
|  | Others |  | 10.81% |  | 21.97% |  |  | 21.57% |  |  | 10.42% |  |
| Co-residence | No |  | 35.71% |  | 38.64% | 0.42 |  | 31.37% | 0.54 |  | 35.42% | 0.97 |
|  | Yes |  | 64.29% |  | 61.36% |  |  | 68.63% |  |  | 64.58% |  |
| Self-rated health | | 3.5 (1.07) |  | 3.23 (1.09) |  | 0.007 | 3.51 (1.15) |  | 0.99 | 3.19 (1.27) |  | 0.33 |
| Number of chronic Conditions | | 1.85 (1.43) |  | 1.9 (1.39) |  | 0.99 | 1.57 (1.65) |  | 0.99 | 1.89 (1.61) |  | 0.99 |
| Perceived difficulty | | 4.76 (2.48) |  | 4.68 (2.71) |  | 0.99 | 5.02 (2.79) |  | 0.99 | 5.46 (3.23) |  | 0.46 |
|  | Financial difficulty | 1.33 (0.88) |  | 1.56 (1.16) |  | 0.02 | 1.59 (1.19) |  | 0.52 | 1.58 (1.27) |  | 0.62 |
|  | Emotional difficulty | 1.9 (1.25) |  | 1.66 (1.24) |  | 0.06 | 1.82 (1.29) |  | 0.99 | 2.06 (1.37) |  | 0.99 |
|  | Physical difficulty | 1.52 (1.13) |  | 1.46 (1.07) |  | 0.99 | 1.61 (1.22) |  | 0.99 | 1.81 (1.45) |  | 0.56 |
| Social isolation | | 3.97 (1.66) |  | 4.09 (1.65) |  | 0.99 | 4.49 (2.02) |  | 0.23 | 4.71 (1.84) |  | 0.03 |
|  | Social disconnectedness | 2.13 (1.3) |  | 2.28 (1.21) |  | 0.77 | 2.47 (1.38) |  | 0.44 | 2.75 (1.28) |  | 0.009 |
|  | No friend/family to talk |  | 16.02% |  | 17.05% | 0.72 |  | 19.61% | 0.51 |  | 22.92% | 0.22 |
|  | No visit to friend/family |  | 18.15% |  | 28.79% | 0.001 |  | 33.33% | 0.009 |  | 33.33% | 0.01 |
|  | No church participation |  | 44.79% |  | 28.03% | <0.001 |  | 33.33% | 0.12 |  | 52.08% | 0.33 |
|  | No club participation |  | 63.13% |  | 71.59% | 0.02 |  | 74.51% | 0.11 |  | 83.33% | 0.005 |
|  | No volunteering |  | 71.24% |  | 82.58% | 0.001 |  | 86.27% | 0.02 |  | 83.33% | 0.07 |
|  | Loneliness | 1.84 (0.92) |  | 1.82 (0.98) |  | 0.99 | 2.02 (1.12) |  | 0.99 | 1.96 (1.07) |  | 0.99 |
| Depression | | 2.99 (1.33) |  | 3.16 (1.33) |  | 0.62 | 3.45 (1.63) |  | 0.13 | 3.13 (1.44) |  | 0.99 |
| Anxiety | | 3.17 (1.39) |  | 2.82 (1.25) |  | 0.005 | 3.19 (1.74) |  | 0.99 | 3.25 (1.52) |  | 0.99 |

Supplementary Table 2. Correlation coefficients between variables

|  | 1 | 2 | 3 | 4 | 5 | 6 | 7 | 8 | 9 | 10 | 11 | 12 | 13 | 14 |
| --- | --- | --- | --- | --- | --- | --- | --- | --- | --- | --- | --- | --- | --- | --- |
| 1 Age | -- |  |  |  |  |  |  |  |  |  |  |  |  |  |
| 2 Gender (women) | -0.11*** | -- |  |  |  |  |  |  |  |  |  |  |  |  |
| 3 Education | -0.14*** | 0.08* | -- |  |  |  |  |  |  |  |  |  |  |  |
| 4 Marital Status | -0.36*** | 0.09** | -0.02 | -- |  |  |  |  |  |  |  |  |  |  |
| 5 Co-residence | 0.24*** | -0.08* | -0.13*** | -0.09* | -- |  |  |  |  |  |  |  |  |  |
| 6 Self-rated health | -0.01 | -0.04 | 0.16*** | -0.03 | -0.08* | -- |  |  |  |  |  |  |  |  |
| 7 Number of chronic conditions | 0.38*** | 0.04 | -0.13*** | -0.16*** | 0.15*** | -0.34*** | -- |  |  |  |  |  |  |  |
| 8 Financial difficulty | -0.16*** | 0.07* | 0.02 | 0.07* | 0.02 | -0.13*** | -0.01 | -- |  |  |  |  |  |  |
| 9 Emotional difficulty | -0.10** | 0.16*** | 0.11** | 0.03 | 0.07* | -0.15*** | <0.01 | 0.37*** | -- |  |  |  |  |  |
| 10 Physical difficulty | -0.02 | 0.13*** | 0.05 | 0.02 | 0.06 | -0.28*** | 0.10** | 0.31*** | 0.29*** | -- |  |  |  |  |
| 11 Functional limitations | -0.05 | 0.10** | <0.01 | 0.04 | 0.07* | -0.06 | <0.01 | 0.14*** | 0.17*** | 0.23*** | -- |  |  |  |
| 12 Dementia status | <0.01 | 0.12*** | <0.01 | 0.10** | 0.02 | -0.09** | 0.01 | 0.09** | 0.14*** | 0.15*** | 0.32*** | -- |  |  |
| 13 Social isolation | -0.08* | -0.01 | -0.15 | 0.24*** | 0.18*** | -0.26*** | 0.04 | 0.17*** | 0.19*** | 0.22*** | 0.14*** | 0.12*** | -- |  |
| 14 Depression | 0.04 | 0.03 | -0.04 | 0.06 | 0.10** | -0.26*** | 0.13*** | 0.16*** | 0.25*** | 0.19*** | 0.17*** | 0.19*** | 0.25*** | -- |
| 15 Anxiety | -0.05 | 0.07* | <0.01 | 0.04 | 0.04 | -0.25*** | 0.11** | 0.15*** | 0.27*** | 0.22*** | 0.08* | 0.07* | 0.26*** | 0.39*** |
| * p < 0.05, ** p < 0.01, *** p < 0.001 | | | | | | | | | | | | | | |
